# A Rough Guide to Metabolite Identification Using High Resolution Liquid Chromatography Mass Spectrometry in Metabolomic Profiling in Metazoans

**DOI:** 10.5936/csbj.201301005

**Published:** 2013-02-15

**Authors:** David G. Watson

**Affiliations:** a Strathclyde Institute of Pharmacy and Biomedical Sciences, 161, Cathedral Street, Glasgow G4 0RE, United Kingdom

**Keywords:** Elemental composition, mass deviation, isotope peaks, MS^2^

## Abstract

Compound identification in mass spectrometry based metabolomics can be a problem but sometimes the problem seems to be presented in an over complicated way. The current review focuses on metazoans where the range of metabolites is more restricted than for example in plants. The focus is on liquid chromatography with high resolution mass spectrometry where it is proposed that most of the problems in compound identification relate to structural isomers rather than to isobaric compounds. Thus many of the problems faced relate to separation of isomers, which is usually required even if fragmentation is used to support structural identification. Many papers report the use of MS/MS or MS^2^ as an adjunct to the identification of known metabolites but there a few examples in metabolomics studies of metazoans of complete structure elucidation of novel metabolites or metabolites where no authentic standards are available for comparison.

## 1. Introduction

This article examines the application of liquid chromatography high resolution mass spectrometry (LCHRMS) in metabolomic profiling in relation to metabolite identification. The review focuses on metabolomics in metazoans since the issues with regard to identification are different to the issues with regard metabolite identification in plants and microbes, but not necessarily simpler. On the one hand the diversity of chemical structures in plants and microbes is huge in comparison with metazoans but on the other these metabolites can often be obtained in large quantities and are amenable to isolation and characterisation by nuclear magnetic resonance spectroscopy (NMR). Identification of metabolites can be a problem in mass spectrometry based metabolomics [1–7] but sometimes the issue is made to seem more difficult than it actually is. If the goal of a metabolomics study is to monitor well characterised metabolites for instance in disease monitoring, then low resolution mass spectrometry might be more appropriate since the target compounds are known and tandem mass spectrometry is a method which is highly standardised with regard to being able to obtain quantitative accuracy. Some research groups have set up more specific biomarker screens based on low resolution tandem mass spectrometry where a large number of standard compounds have been used to standardise the methods [8–13]. However, for many research groups biomarker discovery remains a more general screening operation. Gas chromatography mass spectrometry (GC-MS) remains a widely used general screening process and is very effective for the wide range of compounds which are either volatile or can be rendered volatile through derivatisation [14–16]. It has the advantage that capillary gas chromatography is inherently a much higher resolution separation technique than liquid chromatography and thus there are fewer issues with the separation of isobaric or isomeric compounds. In addition the electron impact ionisation spectra produced by GC-MS provide a complex fingerprint which, when matched against library spectra, can differentiate between closely similar compounds such as isomers [1]. The limitation of GC-MS is that there are a large number of compounds within metabolomes which are not volatile or are unstable at the high temperatures required for GCMS. Thus this article focuses on how metabolites can be identified by using LCHRMS.

## 2. Identification in Mass Spectrometry

Although there are thousands of metabolites in biological systems the level of the challenge of identifying them can immediately be reduced if the interest is purely in identifying those that change in the system under investigation. The numbers of metabolites changing in response to for instance diseased vs healthy controls may be only tens of metabolites rather than thousands [16–30]. These are the compounds which hold the most interest with regard to identification since they might be used to support a particular hypothesis or diagnose a particular disease. The metabolomics standards initiative indicated different levels of identification of compounds in mass spectrometry [31, 32].

## 3. Elemental Composition

The most basic level of identification obtained using mass spectrometry is the molecular weight of a metabolite. When high resolution mass spectrometry is used the accurate molecular mass obtained can be assigned to a particular molecular formula. Experimentally measured masses are rarely exactly the same as a proposed molecular formula, so a formula will be assigned by software which has a mass deviation from the exact mass measured in ppm (ppm = (deviation of measured mass in atomic mass units/exact molecular weight)x10^6^). The size of the deviation impacts on the confidence with which an elemental formula can be assigned to a mass. The degree of mass deviation depends on the type of ion separation method used to generate the mass spectrum. There are three major types of mass spectrometer used in high resolution measurements in metabolomics: Time of flight mass spectrometers (TOFs), ion cyclotron resonance mass spectrometers and Orbitraps. Mass accuracy performance is approximately as follows: TOF instruments *ca* 1-2 ppm, Orbitraps ca 1 ppm, ion cyclotron resonance *ca* 0.1 ppm [1, 34–40]. A more detailed discussion of these instruments can be found elsewhere [34, 39, 40]. In addition, optimal performance of an instrument depends on the instrument having been recently calibrated against compounds of known molecular weight and on the choice of suitable lock masses. A lock mass is an exact mass which is used to correct for any instrument drift during an analysis. Often in TOF instruments a compound which is to be used as a lock mass is continuously infused into the mass spectrometer ion source during a run. Alternatively the lock mass may be one or several background ions which are present throughout the analytical run such as ions generated from the solvents used in the chromatographic separation which are continuously being introduced into mass spectrometer source along with the ions of interest. When working with mass accuracy at < 3 ppm there is generally no difficulty in separating compounds with the same nominal mass. To get an idea of what ppm separation means in practical terms table 1 lists compounds with masses between 132.05-132.12 with their ppm deviations from the preceding masses in the table. As can be seen in table 1, a high resolution mass spectrometer would have little trouble in distinguishing these isobaric compounds and the main problem, if for instance hydroxyproline was an important marker, would be in distinguishing it from its isomers.

**Table 1:** Isobaric compounds between 132.0-132.2 (taken from the Metlin database)

Compound	Exact Mass	Molecular Formula	▵ppm
Iminoaspartic acid, oxosuccinamate	132.0291	C_4_H_5_NO_4_	-
N-acetylalanine,propionyl glycine, hydroxyproline, aminolevulinic acid, oxoaminopentanoic acid, N-acetyl β-alanine, glutamate semi-aldehyde,	132.0655	C_5_H_9_NO_3_	275
Creatinine, guanidino propionic acid	132.0808	C_6_H_13_NO_2_	116
Leucine, isoleucine, alanine betaine, betaleucine, alloisoleucine, aminocaproic acid.	132.1019	C_4_H_9_N_3_O_2_	160
Guanidinobutanol, carbomyl putrescine.	132.1131	C_5_H_13_N_3_O	85

As the mass of a compound increases the number of possible combinations of elements which can add up to make its exact mass increases. The simplest way to evaluate the impact of the ppm deviation of the measured mass on correct assignment of elemental composition is to look at some examples. However, firstly we must consider which elements we should include in the search. Table 2 lists the exact masses of biologically relevant elements, which are covalently bound in naturally occurring organic compounds, along with their isotopes. The list could be slightly wider but selenium is so rare that is not worth considering as a standard element to search for unless it is particularly abundant and the same is true of boron and silicon which may occur in specific biological systems. Halogens occur only rarely in biomolecules such as iodine in thyroxine, in specific biological systems or as xenobiotics in a particular biological system. Chlorine and bromine are instantly recognisable because of their characteristic isotope patterns. Metal ions are generally not covalently bound to organic molecules, there are obviously some exceptions, although they can contribute adducts to mass spectrometric data. Thus to keep it simple, for the purposes of this short review, it is a safe bet that the vast majority of compounds occurring in a biological system contain H,C,O,N,P,S, in that order of frequency of occurrence. Xenobiotics can confound this view but their occurrence is often idiosyncratic and thus they would not be picked up as a significant difference between a treated and control group unless of course, for instance, a drug treatment regimen was being studied.

**Table 2:** The biologically abundant elements and their isotopes

Element	Mass (%Abundance) Main isotope	Mass (%Abundance)
Electron	e (0.00055)	
Hydrogen	^1^H 1.00783 (100)	^2^H 2.01410 (0.0115)
Carbon	^12^C 12.00000 (100)	^13^C 13.00335 (1.08)
Nitrogen	^14^N 14.00307 (100)	^15^N 15.00011 (0.37)
Oxygen	^16^O 15.99491 (100)	^17^O 16.99130 (0.21)
		^18^O 17.99916 (0.04)
Phosphorus	^31^P 30.97376 (100)	
Sulphur	^32^S 31.97207 (100)	^33^S 32.97146 (0.8)
		^34^S 33.96787 (4.52)
		^36^S 35.96708 (0.02)

## 4. The Assignment of Elemental Composition

Kind *et al* defined a formal set of rules for confirmation of the correct elemental composition for compounds according to their mass spectra [41]. Below these rules are restated simply for use with high resolution masses. These rules are not definitive but they present a simple first pass method for evaluating unknown metabolites.

1. Formula containing any combination of carbon, hydrogen, sulphur and oxygen, not necessarily all of these elements, cannot have an even MW in their protonated or deprotonated form (some compounds are fractionally (< 0.1 amu) below their nominal mass e.g. the negative ion of fructose bisphosphate (C_6_H_13_O_12_P_2_) has a mass of 338.9888 which for the purposes of this rule is regarded as 339).
2. Formulae containing odd numbers of nitrogen atoms have even MWs in their protonated or deprotonated forms.
3. Formulae containing even numbers of nitrogen atoms have odd MWs in the protonated or deprotonated form.
4. Apart from for peptides it would be unusual to find more than 7 nitrogens in a structure.
5. It would be unusual for the number of oxygens to be more than one greater than the number of carbons. The number of nitrogens in a structure would be unlikely to exceed the number of carbons. The sum of nitrogens and oxygens would be unlikely to exceed the number of carbons. This rule does not always apply if there is one or more phosphorus atoms in the composition.
6. It would be unusual to find more than two sulphur or more than three phosphorus atoms in a structure.
7. One phosphorus requires at least four oxygens (usually found with 6 or more) in a formulae, two phosphorus atoms at least seven oxygens, three phosphorus atoms at least nine oxygen atoms.
8. Molecules containing both phosphorus and sulphur are relatively rare.
9. A degree of unsaturation (RDB equiv.) > 20 would be unusual for a low MW metabolite.
10. Assuming the instrument is tuned sufficiently well to give known marker metabolites accurate masses with < 1.5 ppm deviation, elemental compositions > three times the deviation for the first acceptable composition should be rejected. This rule should be applied in an iterative fashion if the first acceptable composition is rejected since it is absent from the databases.

Thus these rules can provide a framework for simply evaluating an elemental composition. It is not likely in practice that the sort of exercise described below would be carried out ahead of a database match. However, if the exact mass was not in the database then they could be useful. They are not perfect but neither are they complex. With these rules in mind it is possible to look at some examples. The restrictions applied to the formulae shown in table 3 in terms of number of elements allowed were C60 H100 N7 O20 P4 S2 with a RDB ≤ 20. The first example is for a compound with a measured at 744.08258 amu in positive ion mode on an Orbitrap Exactive. The high MW means that the possible variations in elemental composition are large.

**Table 3:** Identification of possible elemental compositions for m/z 744.08258

Hit No.	Dev. ppm	RDB	Elemental composition	Fails rule
1	0.013	2	C17 H40 O16 N4 P4 S2	3,4,5,8
2	-0.027	20	C33 H30 O14 P2 S	1,8
3	0.121	16.5	C27 H26 O20 N3 S	Passes
4	-0.202	12.5	C21 H29 O17 N7 P3	Passes
5	0.215	17	C25 H26 O17 N6 P2	3
6	-0.296	12	C23 H29 O20 N4 P S	3,5,8
7	0.430	6.5	C21 H37 O16 N3 P3 S2	8
8	-0.444	15.5	C29 H33 O14 N P3 S	8
9	0.699	20	C30 H32 O5 N6 P4 S2	3,7,8, 10 at the second iteration

Thus there are two possible hits for this compound according to the rules. In practise automatic comparison against a database of accurate masses would be used to assign an identity to a metabolite. There are a number of databases which contain high resolution mass values of metabolites including the human metabolome database [42], KEGG [43], LipidMaps [44], ChemSpider (http://www.chemspider.com/) Metlin [45] and PubChem (http://pubchem.ncbi.nlm.nih.gov/). PubChem is the largest database of chemical compounds available but it contains many synthetic as well as naturally occurring compounds. Manually searching against PubChem returns no matches for hit 3. Searching the composition of hit 4 against PubChem returns 14 hits and only one of these corresponds to a naturally occurring metabolite, NADP+. Another example is a compound with a measured mass in positive ion mode of m/z 613.1598. This example is more challenging since the mass deviation for the metabolite is greater than the example above. The first match in PubChem is for hit 17 which matches oxidised glutathione (GSSG). Below this in the table there are no matches until formula 31 which matches a series of synthetic anti-tumour agents with complex non-biological structures. Hit 33 gives a match to a single compound in PubChem which is a polyphosphate sugar. In theory such structures could occur in nature but this is a synthetic compound. It is unlikely that one would often go through this laborious way of assigning a structure since database matching eliminates any compounds which are not known biomolecules. However, in the case of a complete unknown compound manual checking would be a component in deciding whether or not a hit seemed genuine. It is notable for the two examples given that the most frequently applied rules are the nitrogen rules 2 and 3.

## 5. Isotope Patterns

A high resolution spectrum contains additional information relating to the molecular ion in the form of an isotope pattern (see isotopes listed in table 2). The exactness of the correspondence of the isotope pattern to the theoretical pattern depends on the type of ion separation device used. TOF instruments give isotope ratios closer to the predicted level since there is no requirement to have a cut off level for low abundance ions. Ion trap instruments such as the Orbitrap have to limit trap fill due to space charge effects which produce interference between ions in the trap if it becomes too full. Thus traps have a cut off for low abundance ions to prevent overfilling of the trap with ions which are essentially due to background noise. Software is available for correlating observed against theoretical isotope patterns and is either provided by vendors is available for free download [46]. The example of GSSG discussed above was obtained at high resolution on an Orbitrap but even at 50000 resolution for a large molecule like GSSG resolution between ^15^N, ^17^O and ^13^C peaks is not achieved and the ions sum into a single ion for the M+1 peak.

**Figure 1:**
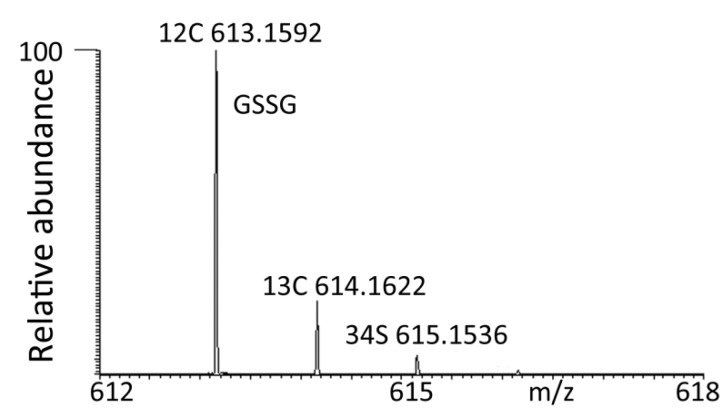
Molecular ion of GSSG extracted from mammalian cells with associated isotope peaks.

In theory GSSG (C20H33O12 N6S2) contains the following isotope patterns calculated from the abundances in table 1.

1. M+1 peak 20 x 1.08 (^13^C) + 0.37 x 6 (^15^N) + 0.21 x 12 (^17^O) = 26.34%. In fact it is present at 19.9% abundance due to the cut off level in the ion trap is thus not an exact match to theory.
2. More specific with regard to confirming identity is the presence of a peak at 615.1572 which is due to the ^34^S isotope (2x 4.52%) which is very characteristic of sulphur containing compounds. The other elements contribute very little at 2 amu higher than the molecular ion and thus the abundance of the [MH]^+^ +2 peak is in theory 9.04% but in the spectrum obtained from the sample it is 5.5%, the effect of the trap cut off becomes more marked for low intensity ions. However, the presence of this ion, despite the deviation from expected intensity, is very characteristic of a sulphur containing compound.

**Figure 2:**
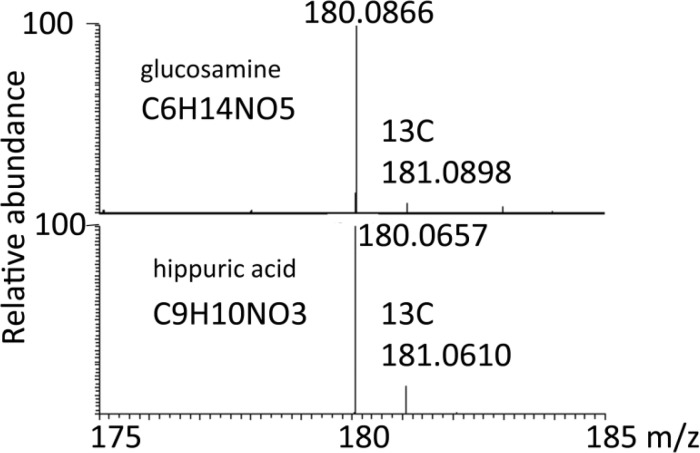
Comparison of the ^13^C-isotope peaks for glucosamine and hippuric acid. The observed intensity values were glucosamine 5.5 % (theoretical 6.8%, the ^17^O peak is resolved) and hippuric acid 9.5% (theoretical 9.9%, the ^17^O peak is resolved).

Isotope ratios can provide a quick check of whether or not a proposed formula makes sense. Figure 2 shows a comparison of the spectra of hippuric acid and glucosamine. These compounds are close in mass but have different numbers of carbon atoms and this can be immediately seen from the height of the ^13^C-isotope peak.

**Table 4:** Identification of possible elemental compositions for m/z 613.1598

Hit No.	Dev ppm	RDB	Elemental Composition	Fails Rule
1	-0.07	20	C34 H31 O6 N S2	2
2	-0.16	7.5	C21 H36 O11 N4 P3	Passes (Not in Pub Chem)
3	0.23	11.5	C27 H33 O14 S	Passes (Not in PubChem)
4	0.24	17	C26 H27 O9 N7 S	2
5	-0.26	12.5	C22 H30 O9 N8 P S	8
6	-0.28	7	C23 H36 O14 N P S	2,8
7	0.34	12	C25 H33 O11 N3 P2	2
8	0.36	17.5	C24 H27 O6 N10 P2	4,7
9	-0.46	16	C28 H34 O3 N5 P3 S	2,7
10	-0.57	15.5	C30 H34 O6 N2 P S2	8
11	0.60	1.5	C21 H44 O10 P3 S2	8
12	0.60	7	C20 H38 O5 N7 P3 S2	2,8
13	-0.78	8	C18 H33 O9 N9 P2 S	2,8
14	-0.78	2.5	C19 H39 O14 N2 P2 S	8
15	0.80	3.5	C14 H34 O11 N10 P S2	4,5,8
16	0.85	16.5	C29 H30 O11 N2 P	Passes (Not in Pubchem)
17	-0.88	7.5	C20 H33 O12 N6 S2	Passes GSSG
18	-1.06	16.5	C25 H31 O N10 P2 S2	4,7,8
19	-1.08	11	C26 H37 O6 N3 P2 S2	2,7,8
20	1.11	11.5	C24 H35 O5 N6 P2 S2	7,8
21	-1.29	3.5	C14 H36 O9 N10 P3 S	4,8
22	1.30	8	C18 H31 O11 N9 S2	2,4
23	-1.40	3	C16 H36 O12 N7 P S2	2,8
24	1.40	3	C17 H37 O13 N5 P2 S	2,8
25	-1.58	6.5	C22 H40 O6 N4 P3 S2	7,8
26	1.61	16	C28 H32 O5 N5 P S2	2,8
27	-1.65	13.5	C20 H25 O13 N10	4,5
28	1.73	11	C27 H38 O7 N P3 S	2,8
29	1.73	16.5	C26 H32 O2 N8 P3 S	7,8
30	-1.84	11.5	C27 H35 O12 P2	Passes (not in PubChem)
31	-1.96	16.5	C28 H29 O10 N4 S	Passes (in PubChem)
33	2.02	2.5	C20 H40 O15 P3	Passes (in PubChem)
39	2.53	12.5	C23 H31 O10 N6 P2	Passes (not in PubChem)
40	2.79	2	C19 H42 O9 N3 P3 S2	2,8, 10 at second iteration

## 6. Misidentification of Isotope Peaks

It is worth noting that isotope peaks can cause some false hits in terms of identification. Data extraction software can be used to filter out isotope peaks but this is a double edged sword since if the isotope peak matches a genuine compound then the genuine hit would be lost unless there was clear chromatographic separation. Returning to the GSSG ^34^S isotope peak which has a mass 615.1536, searched against a database this give a match to within -1.6 ppm for cytidine monophosphate N-acetylneuraminic acid (KEGG entry C00128).There are probably numerous examples of this type of confusion. Two other examples are: Methyl dihydropterin (m/z 178.0723) due to the acetonitrile adduct of hypoxanthine and deoxyribose phosphate (m/z 215.0331) due to a ^35^Cl^-^ adduct of a hexose.

## 7. Identification of isomers

**Figure 3:**
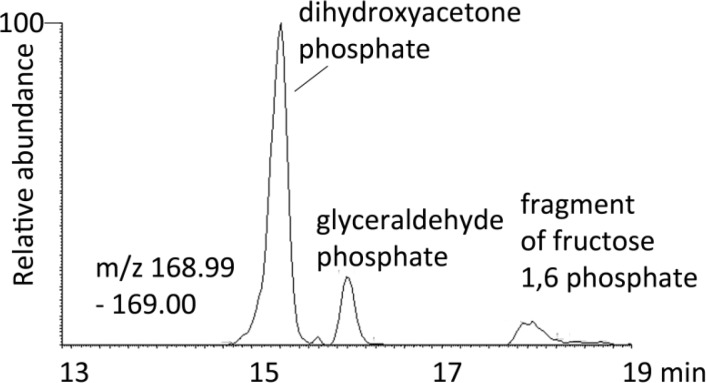
Extracted ion trace for dihydroxyacetone phosphate and glyceraldyde phosphate extracted from mammalian cells separated on a ZICpHILIC column.

The most common problem of identification in high resolution mass spectrometry is in the identification of isomers. There are two strategies available for isomer identification they can either be separated chromatographically and matched against standards or they can be differentiated by fragmenting their molecular ions to produce a mass spectrum where the fragments can be used to characterise the molecule of interest. The most reliable method for characterising isomers is to achieve chromatographic separation since the fragments derive from MS/MS or MS^2^ are often not definitive and if the two isomers overlap chromatographically this will confuse the issue still further. However, setting up a chromatographic method to separate isomers relies on having authentic standards of the molecules of interest, although retention time prediction on the basis of proposed structure can also be helpful [47]. Figure 3 shows an extracted ion trace for dihydroxyacetone phosphate (DHP) and glyceraldehyde phosphate (GP) extracted from a mammalian cell culture on a ZICpHILIC column which produces separation of these two isomers. The additional peak in the trace with the same elemental composition is formed by low level fragmentation of fructose 1,6-diphosphate (F1,6P)in the mass spectrometer. F1,6P is the biosynthetic precursor of DHP and FP.

**Figure 4:**
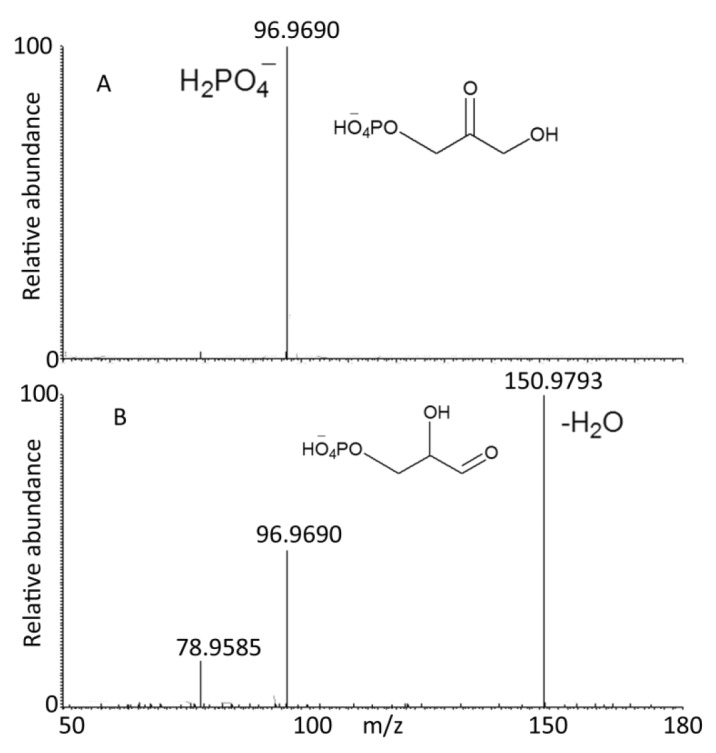
MS^2^ spectra of DHP and GP in negative ion mode (CID 35 V).

In fact DHP and GP can be distinguished by MS^2^ since GP is able to readily eliminate water as DHP cannot (Figure 4).

**Figure 5:**
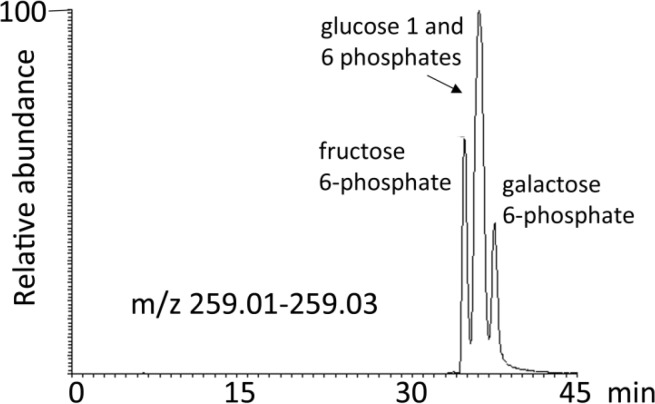
Extracted ion chromatogram for standard sugar phosphates on a 250 mm 4.6 mm Cogent silica C column showing incomplete resolution sugar phosphates.

In contrast figure 5 shows a mixture of sugar phosphates partially separated on a Cogent Silica C column. In the absence of chomatographic separation between isomers then it is necessary to determine whether or not fragmentation can distinguish between isomeric compounds. In case of sugar phosphates there are some differences between tandem MS or MS^2^ spectra [48] of the compounds but the most reliable method for distinguishing between these isomers would be chromatographic resolution. There is only one commonly available method which can chromatographically separate common sugar phosphates such as glucose and fructose 1 and 6 phosphates and that is ion chromatography carried out at high pH. This method exploits the differences in the pKa values of the different hydroxyl groups in the sugars; the hydroxyl groups are appreciably ionised at pH 14 [49].

## 8. Tandem MS and MS^2^ Spectra

Distinguishing between isobaric compounds is much easier than distinguishing between isomers.

**Figure 6:**
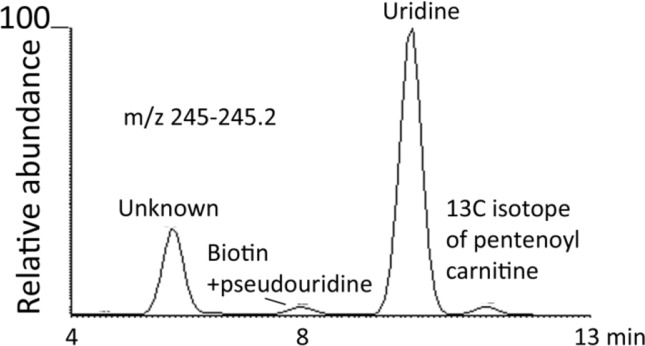
Extracted ion trace over the range m/z 245.0-245.2 of metabolites in human urine.

**Figure 7:**
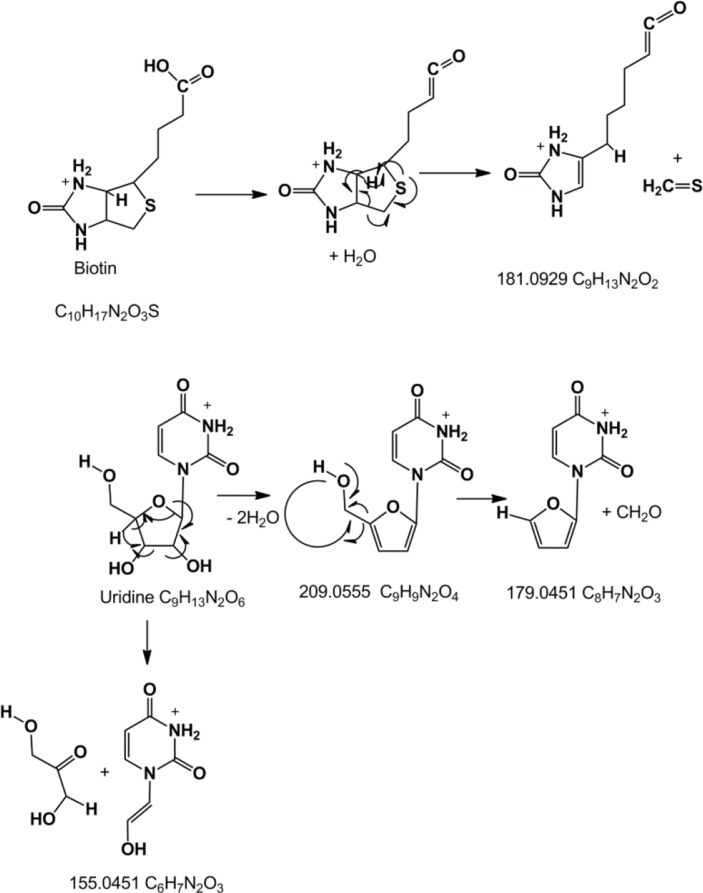
Major MS^2^ fragmentation pathways for biotin and uridine.

**Figure 8:**
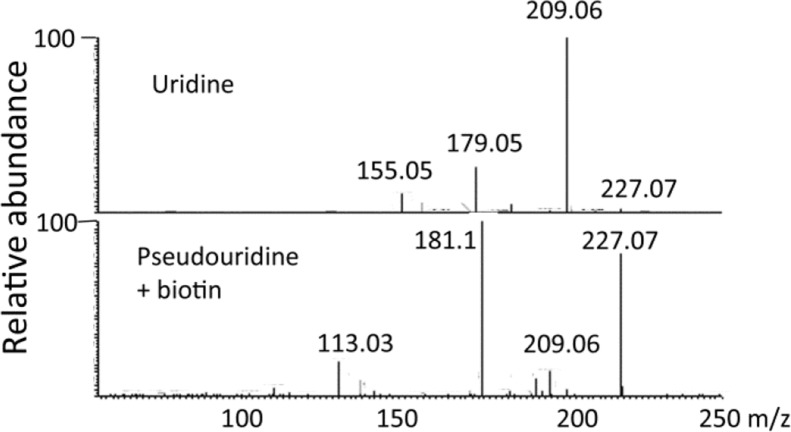
MS^2^ spectra of A Uridine B Pseudouridine + biotin.

Figure 6 shows an extracted ion trace for metabolites in human urine in the range 245.0-245.2 amu. There are four major metabolites evident in this range. The molecular ion composition and MS^2^ data for these compounds is shown in table 5. Two of the exact masses match compounds in Pubchem and other databases and as can be seen in table 5 they give completely different spectra. Many of the rules for fragmentation in mass spectrometry derive electron impact spectra. There have been no definitive rules determined for likely fragmentation pathways by tandem MS or MS^2^ and it is only presumed that similar fragmentation rules apply as were determined for EI conditions. As can be seen in figure 7, where the fragmentations of biotin and uridine are rationalised, fragmentation is not necessarily straightforward. In the present case it is possible to explain the fragmentation but that is with knowing what the compounds are derived from a database match to the elemental composition. This would be less straightforward for a completely unknown structure. Thus mass spectra are often used as a fingerprint without any attempt at interpretation. However, with no database match and no authentic standard to match against a fingerprint is no use. Another problem is indicated in figure 8 where the mass spectra of uridine and pseudouridine in urine are compared. Both compounds are RNA metabolites but pseudouridine is much less abundant in RNA and thus at lower levels in urine. The exact mass of pseudouridine is very close to that of biotin which more or less overlaps with it chromatographically and the more abundant ions in the spectrum are due to biotin. However, it is possible to see fragment ions at 227.0662 due to loss of water from pseudouridine and there is also a peak 113.03 due to loss of the ribose moiety. If pseudouridine was unknown if might be easy in the current example to confuse its MS^2^ spectrum with that of biotin. The metabolite at 5.8 min did not give a match to the database and its fragment ion was not helpful with regard to understanding the structure, it might be that MS^3^ would help to elucidate the structure of the fragment ion but the structure is quite unusual in being highly unsaturated. The metabolite at 11.2 min did not return a sensible elemental composition within a ± 3 ppm window; however, on closer inspection it is the ^13^C-isotope peak of a metabolite at m/z 244.1542 which is probably pentenoylcarnitine since its shows loss of 59 amu in its MS^2^ spectrum which is typical of carnitines.

**Table 5:** MS^2^ data for compounds in the mass range 245.0-245.2 in human urine

Tr Min.	m/z	Elemental Composition	Database Match	Fragment Ions
5.8	245.0920	C_13_H_13_N_2_O_3_	-	170.0597 (C_11_H_8_NO)
8.0	245.0953	C_10_H_16_ N_2_O_3_S	Biotin	227.0845 (C_10_H_14_ N_2_O_2_S), 181.0929 (C_9_H_13_ N_2_O_2_)
9.9	245.0766	C_9_H_12_N_2_O_6_	Uridine	227.0662 (C_9_H_10_N_2_O_5_), 209.0555 (C_9_H_8_N_2_O_4_), 179.0451 (C_8_H_7_N_2_O_3_), 155.0451 (C_6_H_7_N_2_O_3_).
11.2	245.1575	-	-	186.0840

## 9. Tandem MS and MS^n^ in Metabolomics Studies

Many mass spectrometry based metabolomics studies have based their identification of metabolites on the combination of assignment of elemental composition to a metabolite peak and the matching of retention times to the retention times of standards. This is quite reasonable since this is a very specific method already. However, sometimes standards are not available and if a marker compound is significant it is important to characterise it by using tandem MS or MS^2^. Many papers used fragmentation as additional confirmation of compound identity [16–30] but in these papers there is often no discussion the MS/MS spectra since standards are available. The Metlin data base has recorded MS/MS spectra for many naturally occurring metabolites and xenobiotics (http://metlin.scripps.edu) obtained on an Agilent QTOF instrument. MS/MS spectra vary a great deal according to the instrument used, the collision energy used, the collision gas used and the collision gas pressure. More extensive interpretation of mass spectra is required where there is no standard available. There are surprisingly few papers metabolomics papers, studying higher organisms, where the details of structure elucidation of marker compounds have been reported and even many of these do not give full details such collision energy and the gas used for CID. Table 6 summarises some recent studies where fragmentation was used to elucidate the structure and details of the fragmentation patterns observed were given.

**Table 6:** Some publications containing details of structure elucidation using MS/MS or MS^2^.

Nature of Study	Conditions used/Comments	Ref.
Comparison of xanthuria type I and xanthuria type II	LTQ Orbitrap. MS^2^ helium collision gas. Collision energy 35V. HILIC separation. Four products of aldehyde oxidase were characterised based on the accurate masses of their fragment ions. Interpretation of fragment ions provided.	[50]
Metabolomic effects of activation of pregnane X receptor.	Waters QTOF. RP separation. α-carboxyethyl hydroxychromane β-D-glucoside identified as a novel metabolite of vitamin E attenuated by activation of pregnane X receptor. An authentic standard was synthesised.	[51]
Diagnostic markers for ovarian cancer in human serum.	Waters QTOF, preparative HPLC and Bruker FT-ICR. Initial measurements made using RP separation. The structure 27-nor-5β-cholestane-3,7,12,24,25 pentol glucuronide was elucidated by MS/MS, high accuracy mass measurement and hydrolysis of the glucuronide followed by comparison with a standard.	[29]
Profiles of non-polar metabolites in rat and mouse liver altered in response to alcohol.	Shimadzu IT TOF. RP separation. Several fatty acids, fatty acid esters and cholesterol identified as markers of alcohol consumption. Details given for MS/MS spectra of markers. Comparison made against authentic standards.	[52]
Human serum biomarkers of Onchocersiasis.	Agilent QTOF with CID 20V and RP separation and Bruker FT-ICR. Fourteen biomarkers observed and structures partially or completely determined by MS/MS and high resolution MS.	[53]
Biomarkers of the effects pressure in divers.	Agilent QTOF and RP separation. Over 100 metabolites were altered. Tables provided with their major fragment ions.	[54]
Characterisation of extracellular metabolites of Chinese hamster ovary cells.	LTQ Orbitrap and Waters Synapt instrument with CID at 6 or 20V and argon collision gas. RP separation. The Orbitrap provided diagnostic ions for a series of glutamyl peptides. The Synapt system was used to isolate glutamyl phenylalanine from co-eluting isobaric compounds which produced interference in its MS/MS spectrum.	[55]
Profiling of metabolites in human CSF.	Agilent TOF 6210, 20eV. RP separation. Twelve metabolites shared between human plasma and CSF were characterised by MS. Tables provided with their major fragment ions.	[56]
Serum biomarkers of hepatocellular carcinoma.	Waters QTOF and RP separation. Details given from MS/MS structure verification of seven marker compounds.	[57]
Identification of metabolites in human urine.	LTQ Orbitrap, 20V CID. RP and PFP column used for separation. Compounds annotated using standards and MS/MS. Details of MS/MS provided in supplementary tables.	[58]
Metabolic map of procainamide metabolism in mice and humans.	Waters QTOF. RP separation. Thirteen urinary metabolites of procainamide including nine novel metabolites were characterised by using MS/MS data.	[59]
Phenotyping of toxicity of Chuanwu in Wistar rats.	Waters QTOF Micro Synapt. RP separation. The MS/MS spectra of a range of novel biomarkers of toxicity are described.	[60]
Determination of glycosphingolipids markers of Fabry's disease.	Waters QTOF CID 20-35V. RP and HILIC separation. MS/MS was used to characterise a series of glycosylated sphingosine markers compounds.	[61]

If MS/MS or MS^2^ spectra do not provide complete structure elucidation it is possible to use MS^n^ on ion trap instruments to fragment the fragment ions produced in MS^2^ experiments further. Of course this may not always work since the fragment ions produced at the first iteration tend to be stable ions. There is a current trend to move towards MS^n^ identification using spectral trees [62–64] but this is perhaps overkill for the identification of routine metabolites where standards are available, however, it is useful where a complete unknown metabolite is being elucidated.

## 10. Other Factors Supporting Compound Identification

Studies where metabolite hits fall into a single or into closely related metabolic pathways generate greater confidence in the identification of the metabolites within those pathways. Random hits standing in isolation from a defined metabolic pathway are less satisfactory. There are examples of metabolomics studies where the metabolites identified as being changed are metabolically related to each other [16, 64–67] but there are many more where the metabolite changes are seemingly random. Another strategy for identifying metabolites is to predict metabolites in a series. For example phenylacetyl glutamine is an abundant urinary metabolite but no other fatty acid conjugates of this type had been described. It was possible explain a number of metabolites in urine through predicting that other conjugates in this series would be present by simply making a synthetic list of metabolites with the appropriate exact mass by combining fatty acid acyl moieties and glutamine [68].

## 11. Conclusion

In this short review I have tried to simplify the process of metabolite ID by breaking it down into discrete steps. When using high resolution mass spectrometry to identify metabolites in conjunction with a database search, most often the major challenges are chromatographic rather than mass spectrometric since it important to ensure that isomeric compounds are separated. It is important to distinguish challenge of working with metabolites in metazoan systems from the more complex challenges of identifying metabolites in plants. The range of metabolites in metazoan systems is much more restricted than that in plants. However, low levels of unknown metabolites are likely to remain difficult to identify since the only definitive technique for full structure elucidation is NMR which requires mg amounts of material, although the use of capillary NMR tubes can reduce this. Surveying the literature there are not that many examples where an unknown metabolite highlighted in a metabolomics screen has been identified by mass spectrometry. This is likely to change as applications of metabolomics increase. However, the lack of clear examples MS/MS or MS^n^ structural ID experiments in metabolomics in metazoans makes it difficult to assess the effectiveness of these methods. It can be particularly difficult to get useful information when dealing with compounds with low intensity signals where clear fragmentation patterns may not be obtained.
